# Association Between Tail-Biting and Intestinal Microbiota Composition in Pigs

**DOI:** 10.3389/fvets.2020.563762

**Published:** 2020-12-09

**Authors:** Nassima Rabhi, Alexandre Thibodeau, Jean-Charles Côté, Nicolas Devillers, Benoit Laplante, Philippe Fravalo, Guillaume Larivière-Gauthier, William P. Thériault, Luigi Faucitano, Guy Beauchamp, Sylvain Quessy

**Affiliations:** ^1^Chaire de Recherche en Salubrité des Viandes - Conseil de Recherches en Sciences Naturelles et en Génie du Canada (CRSV-CRSNG), Faculté de Médecine Vétérinaire, Université de Montréal, Saint-Hyacinthe, QC, Canada; ^2^Faculté de Médecine Vétérinaire, Centre de Recherche en Infectiologie Porcine et Avicole - Fonds de Recherche du Québec - Nature et Technologies (CRIPA-FRQNT), Université de Montréal, Saint-Hyacinthe, QC, Canada; ^3^Agriculture and Agri-Food Canada, Sherbrooke Research and Development Centre, Sherbrooke, QC, Canada; ^4^F. Ménard Inc., Ange-Gardien, QC, Canada

**Keywords:** behavioral disorder, intestinal microbiota, pig, stress, tail-biting, 16S rRNA gene

## Abstract

Tail-biting (TB) in pigs is a serious behavioral disorder. It is an important challenge in swine production as it impacts animal welfare and health and the economics and safety of the pork meat supply chain. To prevent TB, approaches including enrichment material and tail docking are proposed but none are optimal. Nutrition appears to be an important factor in TB behavior, perhaps by modulating the intestinal microbiota (IM). Our aim was to assess the association between TB behavior and IM in pigs through comparisons of IM in groups of biter, bitten and non-biter/non-bitten pigs. Each group composed of 12 pigs was formed at the beginning of the growing/finishing phase based on a target behavior analysis centered on TB behavior for the biter group and a score of damages caused to the tail for the bitten group. Blood and fecal samples were collected from each pig during a TB episode, at time 0, t0, and when the TB episode was considered finished, 4 weeks later, at time 1, t1. Serum cortisol level was determined by ELISA and used as an indicator of stress. The pig's fecal microbiota was analyzed from DNA extracted from freshly collected fecal matter using amplicon sequencing of the V4 hypervariable region of the 16S rRNA gene. Serum cortisol levels were significantly higher in either the biter or bitten pig groups compared to the negative control group (*p* = 0.02 and *p* = 0.01, respectively). The microbiota alpha-diversity was not significantly different between all groups, biter, bitten and negative control. Analyses of beta-diversity, however, revealed a significant difference between either the biter or the bitten group in comparison to the non-biter/non-bitten negative control group in terms of structure and composition of the microbiota. *Lactobacillus* were significantly more abundant in the negative control group compared to the two other groups (*p* = 0.001). No significant difference was revealed between the biter and bitten groups. Quantitative real-time PCR (qPCR) confirmed that lactobacilli were more abundant in the negative control group. Our study indicates that TB behavior is associated with the IM composition in pigs.

## Introduction

Tail-biting (TB) in pigs occurs when a pig bites another pig's tail. The severity of TB can range from light manipulation that causes no injury, and to which the bitten pig may not react, to physical harm where the tail is wounded, and the bitten pig tries to escape. It is a serious behavioral disorder observed on pig farms (1). It is related to cannibalism (1) and can cause pain, infection, stress (2–4) and reduced growth performance (5) in bitten pigs.

In various studies, the percentages of tail-biting range from 2 to 12% (1, 6). At slaughter, the degree of lesions may range from detectable (70%) to severe (1–3%), resulting in partial or total loss of the tail (7–9). In addition to its effects on animal welfare, tail-biting also impacts the economics of the pork meat supply chain due to veterinary treatments (10), and total or partial carcass condemnation at the abattoir (8, 9). Tail docking reduces tail biting 2–4-fold, but it induces pain, stress and may lead to neuromas and infection (6, 11, 12).

Several risk factors are associated with TB. They are often linked to stress at the farm, including handling practices, housing, confinement, poor air quality (7, 10, 13), poor environment enrichment (14, 15), respiratory disorders (16, 17), and nutrition (10). However, insufficient information is available about the possible correlations between these different risk factors, and it is difficult to clearly identify the triggering cause of TB (18).

Biomarkers of stress in pigs include glucocorticoids, alpha-amylase, chromogranin A, testosterone, acute phase proteins, immunoglobulin A and interleukin-18. Cortisol, a glucocorticoid, is the most widely used biomarker. Whereas serum samples contain both protein-bound cortisol and free cortisol, saliva contains only free cortisol (19).

Several recent studies revealed a correlation between pig gut microbiota and animal health and stress (20–22). Interestingly, biter, bitten and control pigs exhibit different blood metabolites, activity of the immune system and stress levels (3, 23, 24). It is tempting to speculate that biter, bitten and control pigs may harbor different intestinal microbiota (IM) composition (18) which can be regulated by nutrition, feed composition, and stress conditions (18, 25).

The aim of this study was to assess the association between TB behavior and IM in pigs. We first aimed to characterize the animal stress response as measured by its serum cortisol level, and second to compare the IM structure and composition between biters, bitten and non-biter/non-bitten negative control pigs, during and after a TB episode at a commercial farm.

## Materials and Methods

### Animals and Housing

All pigs were handled and treated in accordance with the guidelines of the Canadian Council on Animal Care (26). The protocol was approved by the Animal Use Ethics Committee of the Faculté de médecine vétérinaire of the Université de Montréal (Certificate Number 17-Rech-1858). Written informed consent was obtained from the owners for the participation of their animals in this study.

A total of 352 individually ear-tagged pigs (Landrace × Large White hybrid sows sired with Duroc × synthetic hybrid boar), with undocked tail of 8 weeks of age, were randomly distributed into 32 growing-finishing pens (2.06 × 3.35 m = 6.9 m^2^) located in two separate rooms (16 pens/room) at a commercial farm in Ange-Gardien, QC, Canada. The pens were made of a concrete slatted floor with concrete panels to prevent animal contact between pens. No enrichment, beddings, straw, substrates, objects, toys, were provided. Each pen contained a pig hopper feeder and a waterer with a nipple. Animals always had free access to clean food and water. Ambient conditions, ventilation and temperature, were according to standard housing procedures. Each pen housed 11 pigs (6 gilts and 5 barrows; density 1.6 pigs/m^2^). For proper video identification, a 30 cm number ranging from 0 to 10 was painted on the back of each pig. Each number was associated to each pig ear-tag identifier (Supplementary Figure 1).

In accordance with the feeding practices in place at the commercial farm, all pigs were fed in-house formulated granulated diets containing corn, soy, wheat and canola supplemented with amino acids, vitamins, and minerals. The concentrations of the different ingredients varied according to the phase feeding program in place. This consisted of a pre-fattening feed (supplemented with 0.5 kg/ton of salinomycin) for 2.5 weeks, a fattening feed (with 0.5 kg/ton of salinomycin) for 3.5 weeks, a first growth feed (with 0.21 kg/ton of salinomycin) for 3.5 weeks, a second growth feed (with 0.21 kg/ton of salinomycin) for 10 days, and a finishing feed (with 0.15 kg/ton of narasin) (F. Ménard, Inc., Ange-Gardien, QC, Canada) until slaughter. The treatments did not differ among pens. No pig under study was treated for any disease over the course of this study.

### Behavior Assessment and Selection of the Pig Groups

**Figure 1:**
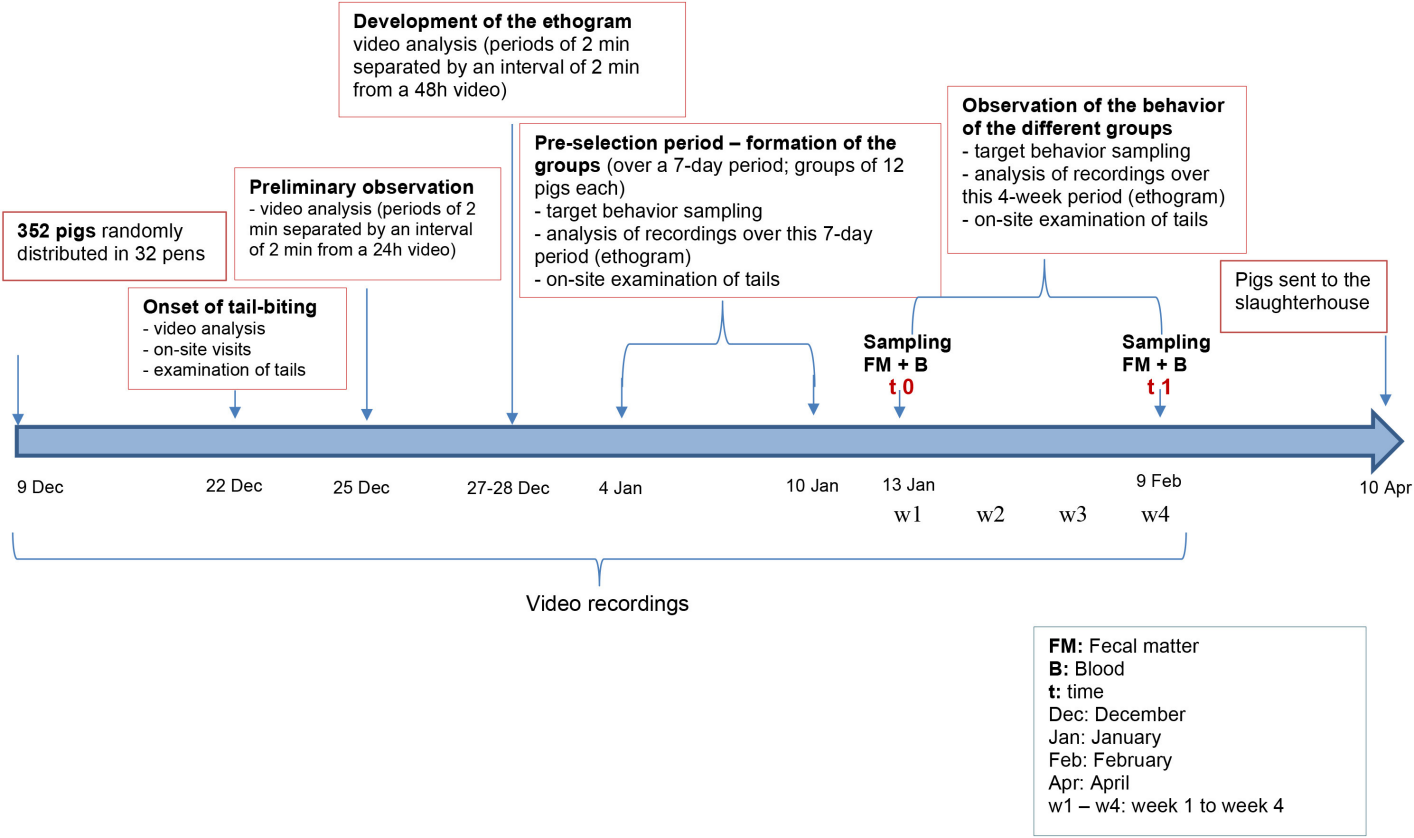
Schematic representation of the timeline of the protocol. Details are presented in section Materials and Methods.

A schematic representation of the timeline of the behavior assessment and the selection of the pig groups is presented in Figure 1. Pig behaviors were recorded following the distribution of the 352 pigs in the 32 pens on video cameras (1,080 p resolution, day/night). Sixteen cameras were installed above the 32 growing-finishing pens under study in a position that allowed the recording of two pens simultaneously (Supplementary Figure 1). Recordings were done 24/24 h throughout the duration of the study and transferred to a computer every 48 h for video analysis of pig behavior. The onset of TB behavior was observed *via* video-analysis, on-site visits and examinations of the tails.

Three days after the onset of TB, a preliminary observation was done to determine the period of the day during which TB occurs. Periods of 2 min, each separated by an interval of 2 min, from a 24-h video recording from four pens in which TB was present, were analyzed.

An ethogram of general pig behaviors was developed. Periods of 2 min, each separated by an interval of 2 min, from a 48-h video recording from five pens in which TB was present, were analyzed. Five different behavior classes were included: rest or awake, eating and drinking, explorative behavior toward the enclosure, harmless social behavior and harmful social behavior. The latter two are presented in Table 1. The harmful social behavior was defined as a pig taking a pen mate's tail in its mouth for at least 5 s without or with a reaction from the victim. This harmful social behavior was categorized as TB.

**Table 1:** Ethogram of pig behaviors recorded during the study [Adapted from Quent (27)]^a^.

**Behavior classes**	**Codes**	**Behavior components**	**Description**
Harmless social behavior—No tail-biting	CTR–	Contact with the tail (without reaction)	The pig touches the pen mate's tail without a reaction from the pig being touched.
	CTR+	Contact with the tail (with reaction)	The pig touches the pen mate's tail with a reaction from the pig being touched. The touched pig is startled or moves away.
	MTR–	Moves the tail (without a reaction)	The pig moves the pen mate's tail without a reaction from the pig being touched.
	MTR+	Moves the tail (with a reaction)	The pig moves the pen mate's tail with a reaction from the pig being touched. The touched pig is startled or moves away.
Harmful social behavior—Defined as Tail-biting	TMR–	Takes the tail in its mouth (without a reaction)	The pig takes a pen mate's tail in its mouth for at least 5 s without a reaction from the receiver.
	TMR+	Takes the tail in its mouth (with a reaction)	The pig takes a pen mate's tail in its mouth for at least 5 s with a reaction from the receiver, who is startled or moves away.

a *Ethogram elaborated from pre-observations of a 48 h video recording from four pens where TB behavior was present*.

**Figure 2:**
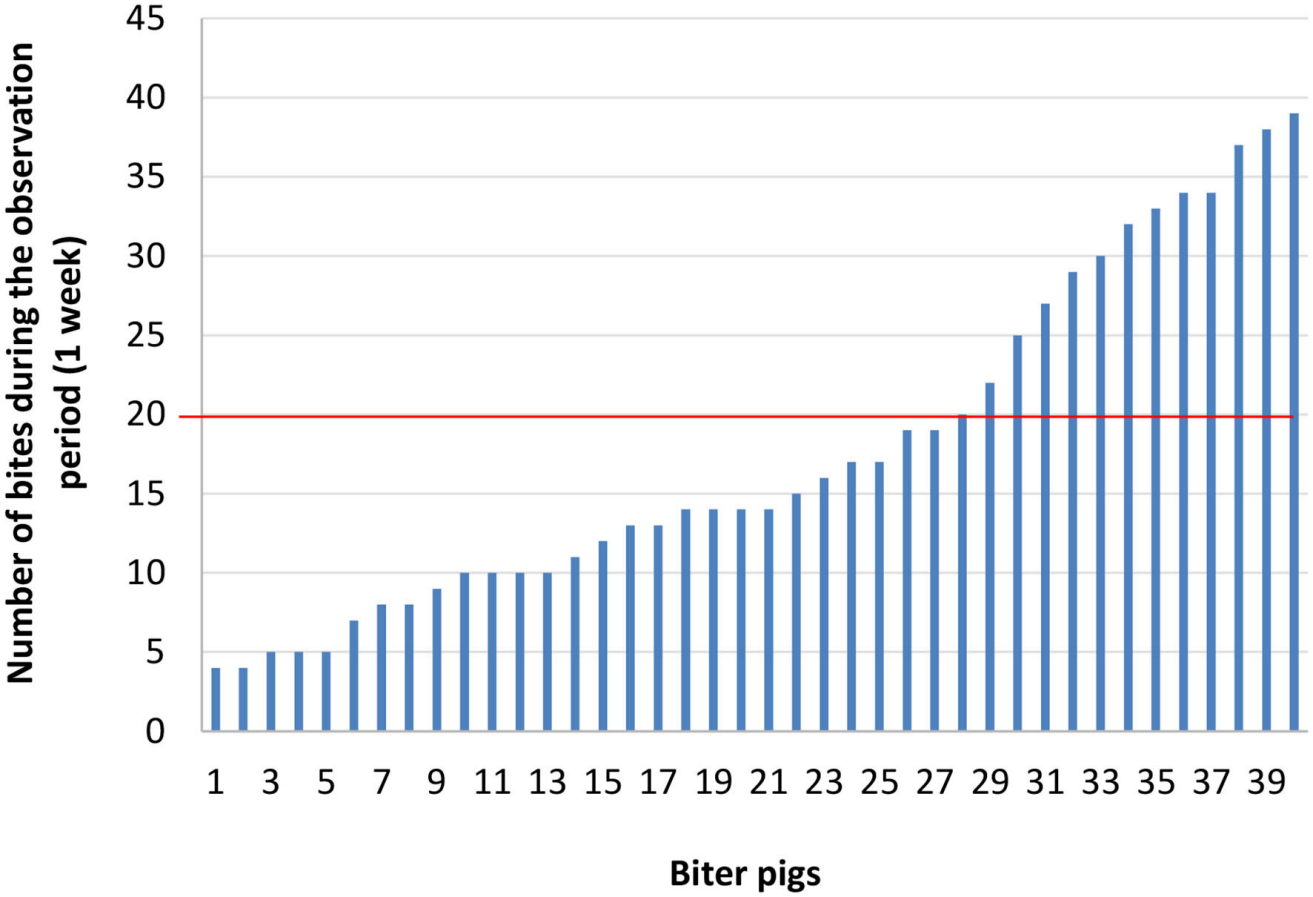
Number of bites for each biter pig over a 7-day observation period during two timeframes: from 10:00 a.m. to noon and from 8:00 p.m. to 10:00 p.m. The red horizontal line indicates the threshold (>20 bites) above which the biter pigs were selected to obtain the number of pigs (12) required for the study.

**Figure 3:**
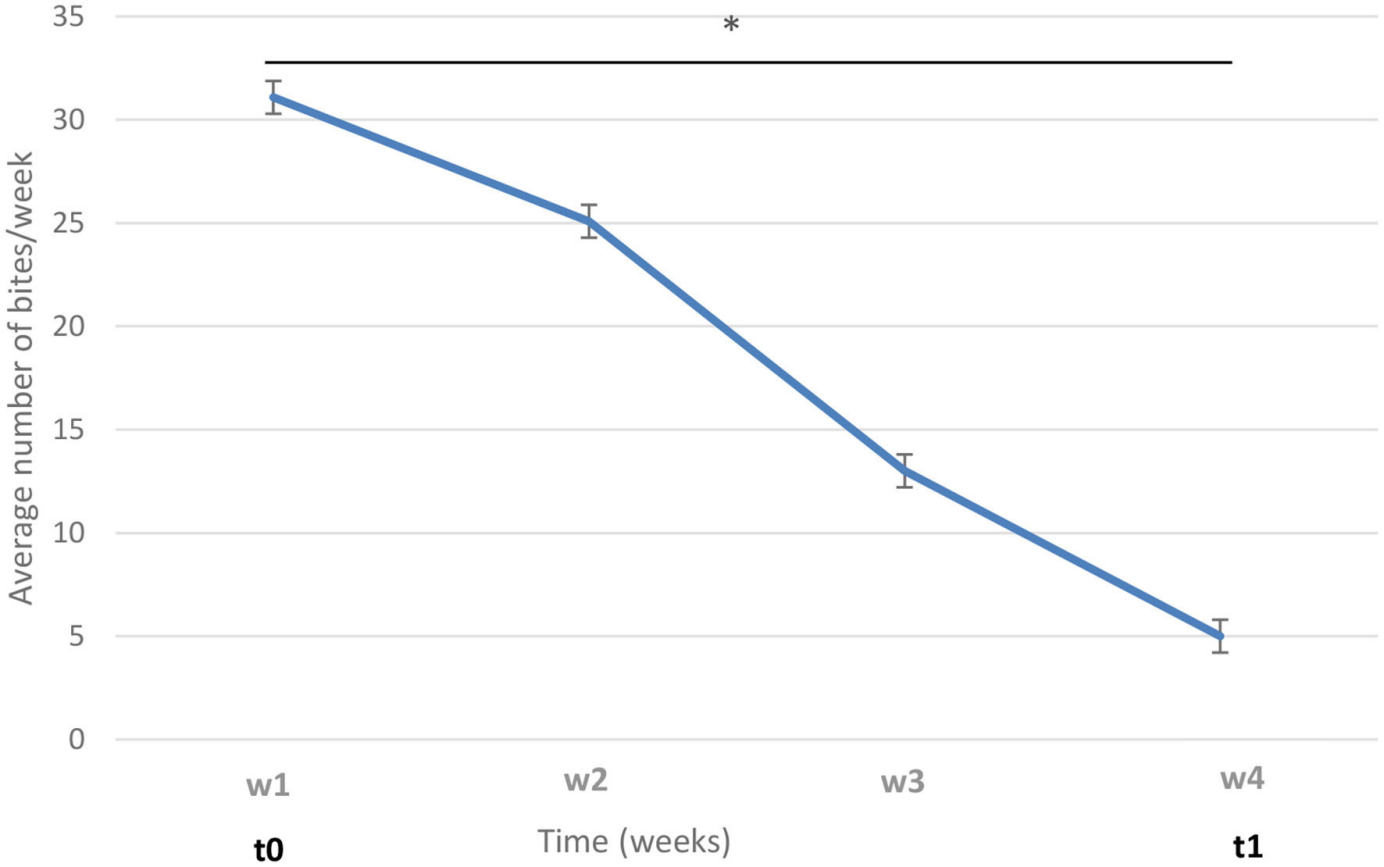
Variation of tail-biting (TB) behavior in biters over time. The average numbers of bites from the 12 biters are shown on a weekly basis, from week 1 to week 4 (w1–w4). t0: time of biter pig group selection; t1: week 4. The vertical lines represent the standard error. The horizontal line indicates statistically significant differences between weeks (*p < 0.0001).

Next, three groups were formed: biter, bitten, and non-biter/non-bitten, based on a combination of the target behavior sampling method (28), the analysis of the recordings of the 10 pens where TB behavior was present over a 7-day period using the ethogram developed above (Table 1), and daily on-site examinations of tails (Supplementary Figure 2). The video analyses were restricted to two timeframes: from 10:00 a.m. to noon and from 8:00 to 10:00 p.m., which corresponded to the two peaks of TB behavior. Forty biter pigs were pre-selected based on total TB behavior frequency ranging from 4 to 39 bites over this 7-day pre-selection period within the two timeframes analyzed (Figure 2). To obtain the targeted sample size of 12 pigs per group, the biters with the highest biting frequency (>20 bites) within this 7-day period and during the two timeframes indicated above, were selected (Figure 2). These 12 biter pigs were used for later analyses of serum cortisol concentration and feces microbiota. For bitten pigs, tail damage was scored from 0 to 3 by a trained observer inside the pen who could touch and examine the tail closely, where 0: no tail damage, no lesion; 1: some small scratches visible on the tail; 2: presence of a small bleeding lesion; and 3: presence of a major bleeding lesion, up to the loss of the tail (28) (Supplementary Figure 2). The pig tail was examined on-site every 2 days in the 10 pens where TB behavior was present. Twelve bitten pigs with a score 2 of tail damage were randomly selected for the later analyses. A third group of 12 pigs randomly selected from three pens where no biter and no bitten was present formed the non-biter/non-bitten negative control group. A fourth group comprising 12 randomly selected non-biter/non-bitten pigs from four other pens where no biter, no bitten, and no non-biter/non-bitten negative control pigs was formed. Chlortetracycline at 1,210 ppm was added to their diet 7 days prior to each sampling date to induce conformational changes in their IM. This fourth group, referred to as ATB (for antibiotics), served solely as a positive control in our later DNA extraction, 16S rRNA gene amplification, sequencing and analysis. At the end of the pre-selection period, the pigs were 12 weeks of age and had been in their growing-finishing pens for 4 weeks (Figure 1). This is referred to as time 0 (t0). All pigs were kept in their original pens. The behavior of all four groups was observed throughout the TB behavior episode over the following 4 weeks using the target behavior sampling, the analysis of video recordings and on-site examination of the tails (Figure 1). The number of bites for each of the 12 biters was computed daily over a 7-day period using video analysis and restricted to the two daily timeframes indicated above. The mean number of bites from the 12 biter pigs was plotted on a weekly basis over this 4-week period referred to as week 1, week 2, week 3, and week 4 (w1, w2, w3, and w4) (Figure 3). The end of week 4 is referred to as time 1 (t1) where TB behavior was considered finished based on the target behavior sampling, the analysis of video recordings and on-site examination of tails as described above (Figure 1).

All pigs were video-monitored throughout the course of this study. Pigs exhibiting health problems or with serious lesions on their body were removed from their respective pens, transferred to a safe pen and treated in accordance with the guidelines of the Canadian Council on Animal Care (26). These pigs were not included in the groups under study.

### Blood and Feces Sampling

A schematic representation of the timeline of the blood and feces sampling is presented in Figure 1. Blood and feces were sampled between 8 and 10 a.m., inside the pen, in the biter, bitten, and non-biter/non-bitten negative control pig groups, during the two different periods referred to as t0 and t1 as described above (Figure 1). No blood sampling was done on the ATB group.

Each pig was restrained with a snare for 1 min by an experienced animal care technician and the blood (~5 ml) collected from the jugular vein by a second experienced animal care technician, using a vacutainer within 30 s. Pigs, biters or bitten, were randomly sampled within a pen before moving to another randomly selected pen. The non-biter/non-bitten negative control pigs were sampled last. The blood samples were kept at room temperature for 2 h to allow blood to clot prior to centrifugation (15 min at 1,000 × g). The serum was transferred to 1.5-mL Eppendorf tubes and stored at −80°C pending cortisol concentration analysis. The quantitative determination of cortisol in serum was done by immunoassay using the Cortisol (Pig) ELISA Kit (Abnova, Taipei, Taiwan) according to the manufacturer's protocol. The minimum detectable concentration of cortisol was 0.2 ng/ml.

Feces sampling was done following blood sampling. A pig was immobilized by a single experienced animal care technician, and fresh fecal material was collected directly from the rectum of the animal by a second technician using fresh clean gloves. A one-gram fraction (from five 200 mg subsamples from the same animal) was frozen immediately in liquid nitrogen and stored at −80°C until DNA extraction and processing.

### DNA Extraction, 16S Ribosomal RNA Gene Amplification, Sequencing and Analysis

Total DNA was extracted from 500 μg of each feces sample according to Thibodeau et al. (29). Briefly, samples were put in tubes containing 0.1 mm glass beads. Bacteria were lysed with 500 ml of lysis buffer (500 mM Tris-HCl, 200 mM EDTA, 1% SDS; Fisher Scientific, Ottawa, ON, Canada) and a FastPrep-24 5G™ High Speed Homogenizer (mpbio, Santa Ana, CA, USA) for 2 cycles of 40 s at 6 m/s. Samples were kept on ice between cycles. DNA was purified using phenol: chloroform: isoamyl alcohol 25:24:1 (Sigma-Aldrich, St. Louis, MO, USA). The phenol traces were removed using chloroform: isoamyl alcohol 24:1 (Sigma-Aldrich). The DNA was precipitated in 90% ethanol for 24 h at −20°C and resuspended in 1 mM Tris-HCl:0.1 M EDTA, pH 8.0. The negative control without feces and the positive control with a known bacterial community (ZymoBIOMICS ™ Microbial Community Standard; Zymo Research, Irvine, CA, USA) were processed in parallel with the fecal samples. The purified DNAs were quantified using a QFX Fluorometer (DeNovix, Wilmington, DE, USA) with Qubit BR reagents (Fisher Scientific). DNA extracts were stored at −80°C.

The hypervariable V4 region of the 16S rRNA gene was amplified for each sample by PCR using the primer pair 515FP1-CS1F ACACTGACGACATGGTTCTACAGTGCCAGCMGCCGCGGTAA and 806RP1-CS2R TACGGTAGCAGAGACTTGGTCTGGACTACHVGGGTWTCTAAT (Invitrogen, Thermo Fisher Scientific, Waltham, MA, USA) (30). The Platinum SuperFi PCR Master Mix (Invitrogen, Thermo Fisher Scientific) was used with 12.5 ng of DNA in a total reaction volume of 25 μl. The amplification was carried out with an initial denaturation step at 95°C for 5 min, followed by 23 cycles at 98°C for 30 s, 55°C for 30 s, and 72°C for 180 s, and a final elongation step at 72°C for 10 min (31). Amplification was confirmed by gel electrophoresis and amplicons sent to the McGill University and Génome Québec Innovation Center (Montreal, QC, Canada) for barcoding and subsequent Illumina Miseq sequencing (250 paired-ends).

All sequences were analyzed using Mothur software version 1.35.5 (32) according to Larivière-Gauthier et al. (33). Taxonomic assignment of the sequences was made using the Ribosomal Database Project (RDP; https://rdp.cme.msu.edu) (34). The sequences with 97% similarity (equivalent to species level) were grouped into operational taxonomic units (OTUs). Alpha-diversity (number of OTUs per sample, Shannon-even and inverse Simpson indices) of fecal samples from pigs of different groups, at t0 and t1 were calculated in Mothur, using a subsample of 32,374 sequences, the lowest number of samples returned in all samples. For beta-diversity analysis, the distance between all samples was measured by the Yue & Clayton and the Jaccard indices using the same subsampling. Structure of the bacterial communities were visualized by a non-metric multidimensional scaling (NMDS) graph, and each combination of two pig groups, biter/negative, bitten/negative, and biter/bitten, was compared at t0 and t1 by the analysis of molecular variance (AMOVA) (35). In addition, a Linear discriminant analysis (LDA) effect size (LEfSe) (36) was used to discover bacterial taxa significantly associated with each group at each sampling time.

### Real-Time Quantitative PCR of Specific Bacterial Populations

To validate and quantify results obtained from sequencing, a quantitative PCR (qPCR) targeting lactobacilli was performed on all samples (37). Standard curves were made from amplicons derived from the control strain *Lactobacillus acidophilus* ATCC 314. Each well-contained 4 μl of Evagreen (MBI Montreal Biotech, Kirkland, QC, Canada), 0.6 μl of forward primer, 0.6 μl of reverse primer, 12.8 μl of water, and 20 ng of DNA. The amplification was done in a LightCycler 96 real-time PCR (Roche Diagnostics, Mannheim, Germany) using the following program: 50°C for 120 s, 95°C for 10 min, 45 amplification cycles of 95°C for 15 s, and 60°C for 60 s, and a final high-resolution melt analysis. The results, the number of gene copies, were expressed in log per ng of DNA.

### Statistical Analysis

A linear model for repeated measures with time as within-subject factor (SAS v9.3, Cary, NC, USA) was used to compare variations in TB across time. For the comparison of the different alpha-diversity indices, a linear model for repeated measures with time as within-subject factor and group as between-subject factor was used. For quantitative PCR results and serum cortisol levels, a linear model was used with group as factor. The AMOVA-test was used to compare the beta-diversity between the different groups (comparison of two groups at a time) using Mothur (38). Statistical-tests were made on comparisons between the following pig groups: biter/negative, bitten/negative, and biter/bitten, at t0 and t1. The alpha level for these comparisons was adjusted downwards with the sequential method of Benjamini and Hochberg (39).

## Results

### Animal Selection

Tail-biting behavior was first observed sporadically in two growing-finishing pens, 2 weeks after the pig's distribution in the pens. The pigs were then 10 weeks of age. Three days later, TB appeared in two additional pens. Video recordings showed that TB was not limited to the periods when the animals were awake but also occurred during the rest periods. Four days later, TB was observed in 10 pens. By then, the pigs had been in the pens for 3 full weeks and were now 11 weeks of age. The following week served as the pre-selection period. By the end of this period, 4 groups were formed: 12 biter pigs from 10 pens, 12 bitten pigs from 8 pens, 12 non-biter/non-bitten negative control pigs from 3 pens, and 12 non-biter/non-bitten ATB pigs from 3 pens to be used as a control in IM analyses (Supplementary Figure 1). Within the 10 pens where TB was observed, 70% of the pigs showed no tail damage (score 0), 13% showed some scratches on their tails (score 1) and 17% showed some small bleeding lesions on their tails (score 2). No pig showed major bleeding lesion on its tail (score 3). Some pigs developed arthritis or showed paralysis or serious lesions on their body (range of 0 to 2 pigs per pen). These were transferred to a safe pen and excluded from the analyses.

### Behavior and Tails Condition

In biter pigs, TB behavior was at its highest at the time of selection and for the next 7 days (time 0, week 1, referred to as t0, w1) and decreased over the following weeks until it was almost unobservable, during the fourth week (time 1, week 4; t1, w4) (Figure 3).

Tails of all bitten pigs healed between week 2 (w2) and week 4 (w4) after selection and showed no new lesion until the end of week 4. Biters were never bitten; they were true biters. Occasionally, video-monitoring showed that some bitten pigs could bite other pigs. These were not included in our group of 12 bitten pigs. The latter were only bitten, never biters. The non-biter/non-bitten negative control pigs never became biters or bitten pigs.

### Variation of Serum Cortisol Concentration

**Figure 4:**
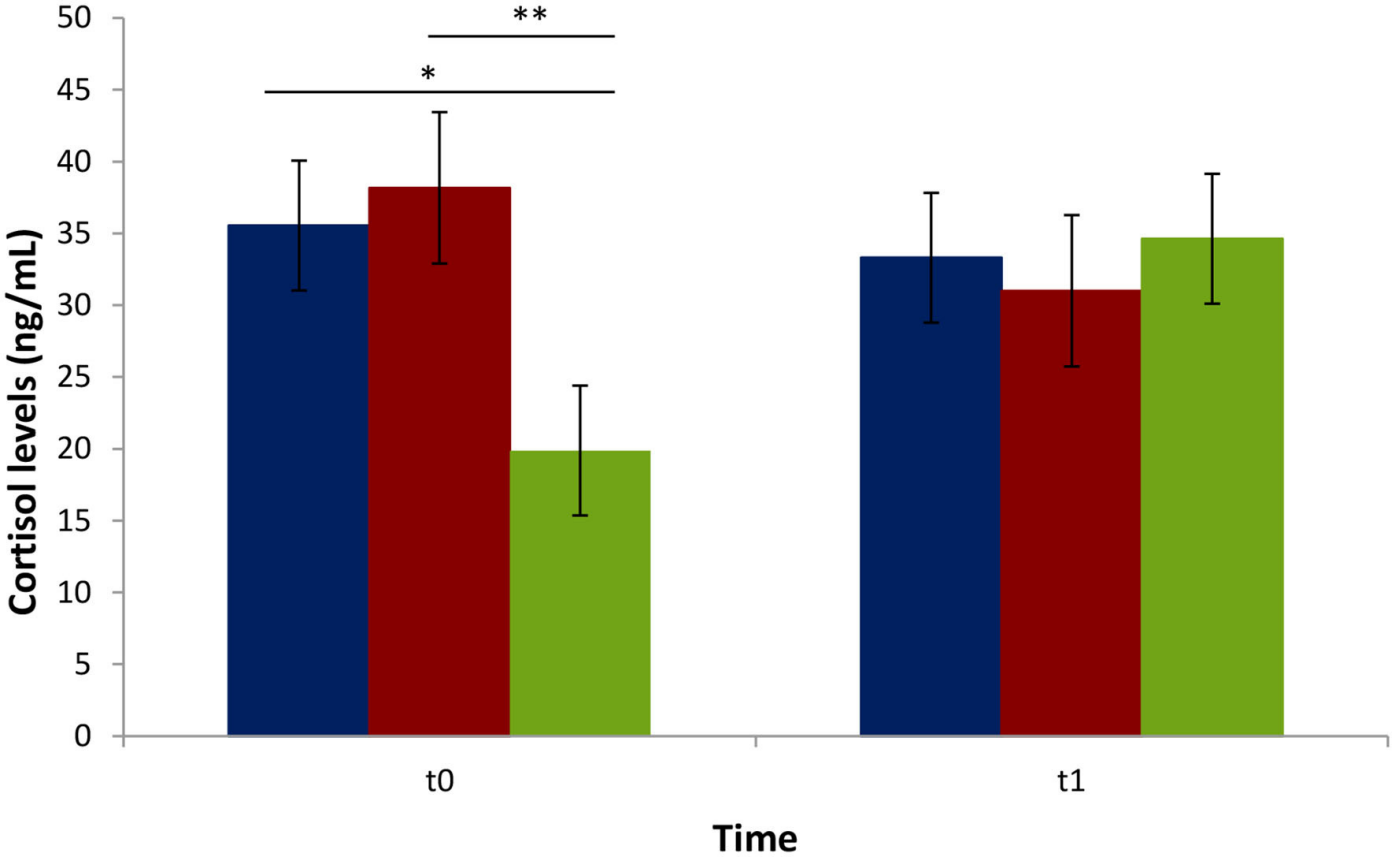
Average cortisol serum levels (ng/mL) of biter and bitten pigs vs. the negative control pigs during a tail-biting episode. t0: time of pig group selection; t1: 4 weeks following selection. Blue: biter pigs; Red: bitten pigs; Green: negative control pigs. The vertical lines represent the standard error. The horizontal lines indicate statistically significant differences between groups (*p = 0.02; **p < 0.01). No difference in blood cortisol levels within the t1 period (*p* > 0.05).

Serum cortisol levels were higher in biter and bitten pigs compared to the negative control pigs at t0 (Figure 4). No significant difference in cortisol levels was observed between the biters and bitten groups at t0 or between all three groups at t1. The cortisol levels were higher in all three groups at t1 compared to the negative control pigs at t0.

### Microbiota Description and Analysis

A total of 6,434,624 sequences were obtained by the sequencing of 47 samples at t0 and 47 samples from the same pigs at t1. The sequences were grouped into 12,810 OTUs using 97% similarity between sequences. The lowest value observed for a sample was 32,374 sequences and 252 OTUs and the highest value was 92,843 sequences and 1 257 OTUs. Most of the sequences were bacterial (6,297,598 sequences, 97.01% of all sequences) and a small fraction was Archean (193,567 sequences, 2.98%). The negative control without feces for DNA extraction and qPCR showed 139 and 4,807 sequences, respectively. Based on the composition of the positive control with a known bacterial community, an acceptable error rate of 0.094% was calculated.

No significant difference in the alpha-diversity indices, OTUs, Shannon-even and inverse Simpson, was revealed between all three groups, biter, bitten and negative control, at t0 and t1 (Table 2).

**Table 2:** Comparison of alpha-diversity indices of the intestinal microbiota of three groups of pigs, biter, bitten and the negative control pigs.

**Periods^a^**						
**Indices**	**Groups at t0**			**Groups at t1**		
	**Biter pigs**	**Bitten pigs**	**Negative control pigs**	**Biter pigs**	**Bitten pigs**	**Negative control pigs**
OTUs	777.85	784.38	791.55	769.28	815.56	834.31
Shannon-even	0.72	0.73	0.711	0.70	0.734	0.73
Inverse Simpson	50.31	55.63	44.22	44.26	53.57	55.21

a *t0, immediately following selection of the pig groups; and t1, 4 weeks later*.

**Figure 5:**
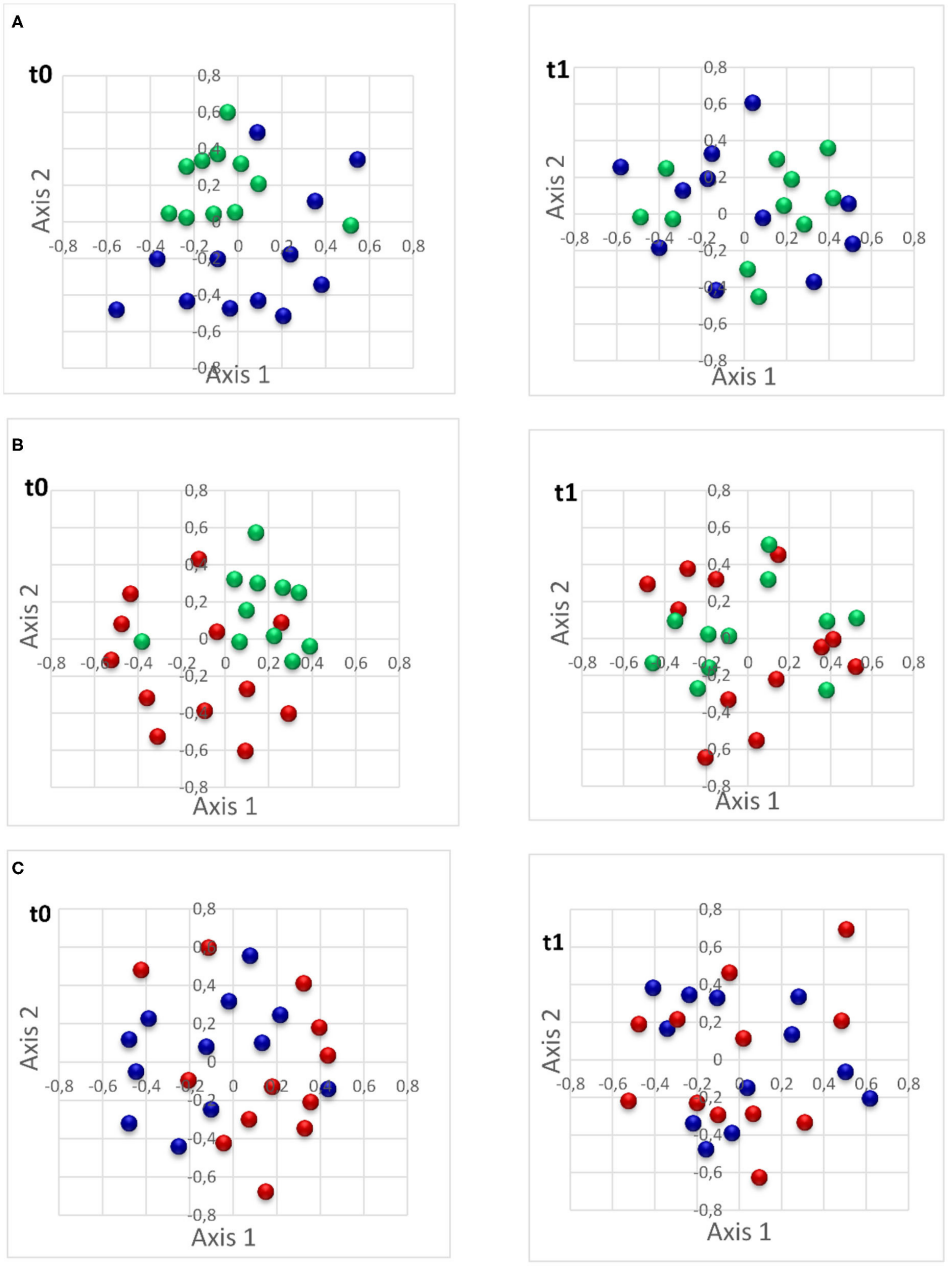
Non-metric multidimensional scaling (NMDS) plot of Yue & Clayton illustrating the comparison of intestinal microbiota of studied pigs. **(A)** The comparison of intestinal microbiota of biter pigs vs. the negative control pigs (at t0 *p* = 0.001; at t1 *p* = 0.24); **(B)**: the comparison of intestinal microbiota of bitten pigs vs. the negative control pigs (t0 *p* = 0.001; t1 *p* = 0.03); and **(C)**: the comparison of intestinal microbiota of biter pigs vs. bitten pigs (t0 *p* = 0.48; t1 *p* = 0.87). t0: time of pig group selection; t1: 4 weeks following selection. Green: negative control pigs; Blue: biter pigs; Red: bitten pigs.

Beta-diversity was compared between the three pig groups at t0 and t1 (Table 3). A significant difference in microbiota structure was observed between the biter and the negative control, and between the bitten and the negative control group, both at t0 with the Yue & Clayton index (Figure 5).

**Table 3:** Comparison of the intestinal microbiota structure of pig groups^a^.

**Time^b^**	**Compared pig groups**		**AMOVA (*p*-value)**	
			**Yue & Clayton index**	**Jaccard index**
t0	Biter	Negative	0.001^*^	0.246
	Bitten	Negative	0.001^*^	0.306
	Biter	Bitten	0.476	0.439
t1	Biter	Negative	0.244	0.318
	Bitten	Negative	0.295	0.55
	Biter	Bitten	0.866	0.46

a *Based on 1,000 subsampling of 32,374 sequences*.

b *t0, immediately following selection of the pig groups; and t1, 4 weeks later*.

* *Statistically significant differences after adjusting the alpha threshold downwards with the sequential Benjamini and Hochberg procedure (39)*.

A LEfSe was performed to identify bacterial taxa indicators of the different groups at each sampling time, t0 and t1. When the biter group was compared to the negative control group at t0, two different genera and 22 different OTUs were significantly (*p* ≤ 0.05) associated with the biter group and three genera and 19 OTUs were associated with the negative control group. In the biter group, the genera *Coprococcus* and *Clostridium* IV showed the highest LDA scores (LDA = 3.3 and 2.69, respectively), while in the negative control group, the genus *Lactobacillus* (LDA = 4.47) and a *Lactobacillus* OTU (LDA = 4.51) showed the highest LDA scores. At t1, two genera, *Roseburia* and *Anaeroplasma* (LDA = 3.23 and 2.91, respectively), and nine OTUs were associated with the negative control group (Supplementary Tables 1, 2).

Likewise, when the bitten group was compared to the negative control group at t0, two genera, *Sphaerochaeta* and *Blautia* (LDA = 3.21 and 2.96, respectively) and 17 different OTUs, most notably *Phascolarctobacterium* (LDA = 3.61) were associated with the bitten group, and two genera, *Lactobacillus* and *Intestinimonas* (LDA = 4.57 and 3.38, respectively) and nine OTUs were associated with the negative control group. At t1, a single genus, *Alistipes* (LDA = 2.91) and five OTUs were associated to the bitten group and five OTUs were associated with the negative control group (Supplementary Tables 3, 4).

The composition and diversity of the intestinal microbiota was not significantly different between the biter and the bitten groups (*p* > 0.05).

When the positive ATB control group was compared with the negative control group, significant differences were found in alpha-diversity indices at t0 (Shannon-even *p* = 0.002 and inverse Simpson *p* = 0.002), and beta-diversities at t0 (Yue & Clayton *p* = 0.001; Jaccard *p* < 0.001) and t1 (Yue & Clayton *p* = 0.028; Jaccard *p* < 0.003) (Supplementary Figure 3). This validates the experimental model and subsequent bioinformatics analysis abilities to measure and identify microbiota modification in the studied animals.

### Real-Time Quantitative Polymerase Chain Reaction

**Figure 6:**
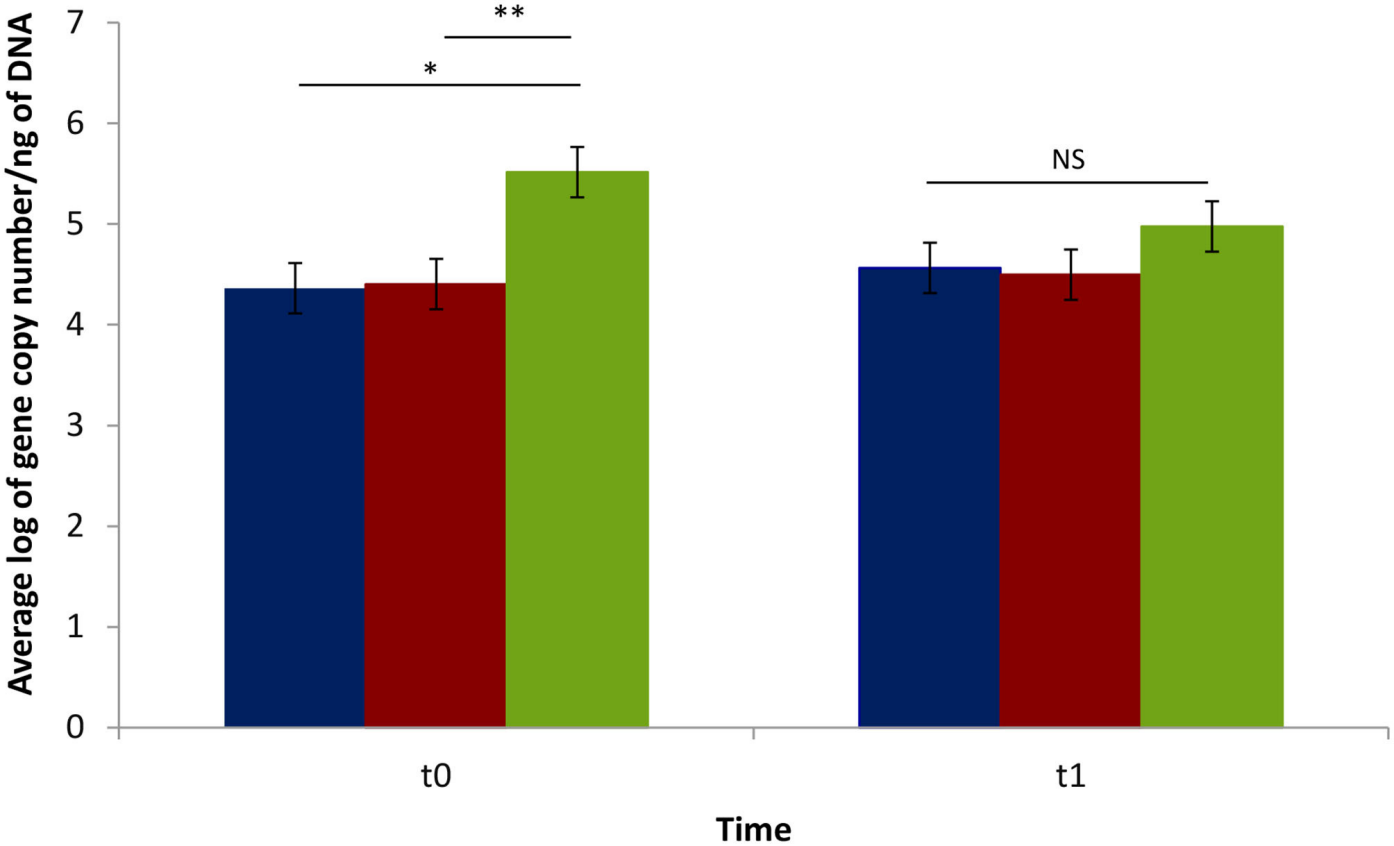
Average log of gene copy numbers of lactobacilli in biter and bitten pig fecal matter samples vs. the negative control pigs during the tail-biting episode. t0: time of pig group selection. t1: 4 weeks following selection. Blue: biter pigs; Red: bitten pigs; Green: negative control pigs. The vertical lines represent the standard error. The horizontal lines indicate statistically significant differences between groups (*p < 0.003; **p < 0.003). NS: No difference in average log of gene copy number/ng of DNA within the t1 period (*p* > 0.05).

A qPCR assay was performed to quantify and validate the results obtained from LEfSe for the genus *Lactobacillus*. A significant difference was observed when comparing either the biter or the bitten pig groups to the non-biter/non-bitten negative control pig group at t0 (during TB), with respective averages of 1.15 and 1.11 log of gene copies per ng of DNA. However, no difference was observed at t1 (Figure 6).

## Discussion

Our aim was to study a possible association between TB and the IM composition in pigs. Part of our study took place in a commercial pig fattening farm where several risk factors associated with TB such as intact tails and slatted floor pens without enrichment (7) were present. The onset of TB 2 weeks after the beginning of the growing period is in agreement with similar studies on pig TB behavior (7, 40–42). No specific event could be identified as triggering the onset of TB. Over the next 3 weeks, TB was present in 10 of the 32 pens included in the study. Consequently, the pigs from the four groups were selected from a small number of pens (≤10), and a pen effect cannot be excluded but was not measured statistically. In our study, however, all pigs were of the same age, kept in similar pens, under same conditions, treated the same manner, fed the same diets, as to minimize pen effects.

Serum cortisol level was used to assess animal stress at t0 and t1. Cortisol level in pigs follows a circadian rhythm and varies according to age, gender, and stress (43). In our study, blood was always sampled between 8 and 10 a.m. to minimize rhythm variations. Cortisol levels were higher in both biter and bitten pigs at t0, when TB behavior was at its peak in selected animals, compared to the non-biter/non-bitten negative control group, presumably a result of increased response to acute stress in pigs (44, 45). Our results for the biter and bitten pigs are in agreement with those of Smulders et al. (46) and Ursinus et al. (47). Ruis et al. (43) showed that cortisol levels decrease between 12 and 20 weeks of age in non-stressed pigs. In our study, however, at t1, cortisol levels were high in all three groups, biter, bitten, and the negative control, compared to the negative control group at t0. The cortisol levels between the biter and bitten pig groups showed no difference either at t0 or t1, indicative that both groups contained stressed animals irrespective of their age. Interestingly enough, TB decreased over time from t0 to t1, in accordance with Ursinus et al. (47).

Central to this study, we assessed a possible association between TB and IM and we generated novel information on the structure and composition of the IM in the biter and bitten pigs. Numerous studies used high throughput sequencing methods to explore the composition of IM in humans (30), rabbits (48), horses (49), and dholes (50). In our study, the structure and the composition of the IM in feces samples of biter and bitten pigs were compared to the non-biter/non-bitten negative controls. A group of pigs treated with antibiotics in order to modify their intestinal microbiota composition and structure served as a control to detect changes in the pig's IM. As expected, the IM of the animals included in this positive control group was different than the animal's IM from the negative control group. This is in agreement with Jernberg et al. (51). We showed that biter or bitten pig groups had different IM between each other and when each was compared to the negative control pig group. To the best of our knowledge, the bacterial genera and OTUs associated respectively to the biter, the genus *Coprococcus*, and bitten pig groups, the genus *Sphaerochaeta* and the *Phascolarctobacterium* OTU, both groups where TB occurred, were not previously associated with behavior disorders.

We also showed that the relative abundance determined by qPCR of *Lactobacillus*, could distinguish biter and bitten groups from the negative control group. It is reported that some *Lactobacillus* species are associated with behavior disorders such as anxiety and depression in both humans (52–54) and mice (55, 56). Conversely, different *Lactobacillus* species are used in probiotics to reduce behavior disorders, such as *Lactobacillus rhamnosus* JB-1 for anxiety and stress (57) or *Lactobacillus helveticus* R0052 for depressive behaviors (58). It would be interesting to test whether the introduction of either or both *Lactobacillus* species in the pig's IM could shorten the TB behavior.

In our study, the pig's microbiota were not analyzed upon their arrival in the growing-fattening pens. It was not possible to determine a possible association between the IM initial composition and apparition of TB or to observe modifications of the IM before t0. Such analysis would contribute to a better understanding of the relationship between TB and IM.

Antimicrobials are useful in the treatment, control and prevention of diseases in pigs and to increase growth performance. They, however, impact the pig IM (59). Our data were obtained with pigs fed formulated granulated diets supplemented with either salinomycin or narasin under the methodology described earlier. Our results cannot be generalized beyond our methodology. It would be interesting to study the effects of various feed modifications, different antimicrobials, the addition of plants extracts, essential oils, probiotics on the pig IM structure and composition, and whether they lead to changes in TB behavior.

## Conclusion

To the best of our knowledge, our results provide the first evidence of a relationship between the occurrence of TB in biters and bitten pigs and IM. Using cortisol level as a marker, we showed that biter and bitten pigs were stressed in comparison to the negative control group. Interestingly, we showed that pigs'IM in the non-biter/non-bitten negative control group had more *Lactobacillus* than in those expressing TB. This is consistent with human and mice studies on the relationship between behavioral disorders and microbiota composition. However, the mechanisms underlying the association between TB and IM are still unknown. Further studies are needed to gain a better understanding of the cause-effect relationship between both.

## Data Availability Statement

The sequencing data were deposited into the Sequence Read Archive (SRA) of The National Center for Biotechnology Information (https://www.ncbi.nlm.nih.gov/sra) and can be accessed via accession number PRJNA634125.

## Ethics Statement

The protocol was reviewed and approved by the Animal Use Ethics Committee of the Faculté de médecine vétérinaire of the Université de Montréal, Certificate Number 17-Rech-1858, in accordance with the Canadian Council on Animal Care guidelines. Written informed consent was obtained from the owners of the commercial farm for the participation of their animals in this study.

## Author Contributions

SQ, NR, AT, BL, and PF planned the overall design of the experiments. NR, ND, BL, and LF planned the animal behavior assessment and blood analysis. AT, NR, and GL-G planned the intestinal microbiota analysis. NR and WPT carried out the experimental work. NR, AT, J-CC, GB, and SQ analyzed the results with contributions from all co-authors. NR, J-CC, SQ and AT wrote the manuscript with contributions from all co-authors. SQ and AT supervised the project. All authors contributed to the article and approved the submitted version.

## Conflict of Interest

BL was employed by F. Ménard Inc. He contributed to the overall design of the experiment at the commercial farm, but was not responsible for data collection, their analysis, and the decision to publish. The remaining authors declare that the research was conducted in the absence of any commercial or financial relationships that could be construed as a potential conflict of interest.
