# Supplementary material for: Association Between Tail-Biting and Intestinal Microbiota Composition in Pigs
Source: Front Vet Sci. 2020 Dec 9;7:563762. doi: 10.3389/fvets.2020.563762 (PMC7756002; doi:10.3389/fvets.2020.563762)
Supplement: Supplementary file 1 [file Data_Sheet_1.pdf]

Supplementary Material

Figure S1. **Schematic representation of animals and housing.**

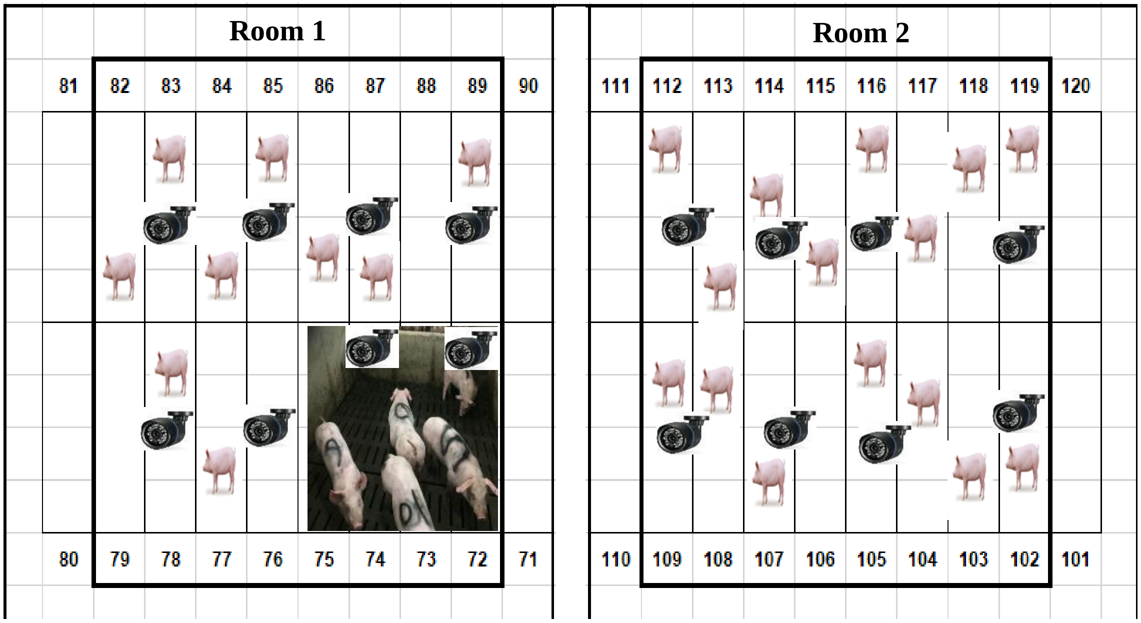

A total of 352 pigs were distributed into two rooms (1 and 2). Each room contained 16 growing-finishing pens (2.06 m x 3.35 m = 6.9 m^2. The pens were made of a concrete slatted floor with concrete panels to prevent animal contacts across pens. No enrichment, beddings, straw, substrates, objects, toys, were provided. Each pen contained a pig hopper feeder and a waterer with a nipple. Eleven pigs (6 gilts and 5 barrows; density 1.6 pigs/m2) were present in each pen. To facilitate the recognition of the pig, a colored number from 0 to 10 was painted on the animal's back. This labeling was supplementary to the ear-tag system already in place at the farm. Their behavior was monitored using 16 surveillance cameras. The numbers 81 to 120 correspond to the pen number.

Biter pigs were from pens #72 (2 pigs), 76, 85, 86, 88, 103, 104, 105, 106, and 107 (2 pigs).

Bitten pigs were from pens #85, 86, 88, 103 (3 pigs), 104, 105 (2 pigs), 106, 107 (2 pigs).

Non-biter/non-bitten negative control pigs were from pens #82, 89 and 112.

Non-biter/non-bitten pigs treated with antibiotics (ATB), used as a control in DNA extraction, 16S rRNA gene amplification, sequencing and analysis, were from pens #84, 109, and 118.

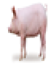	Pig
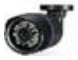	Video camera
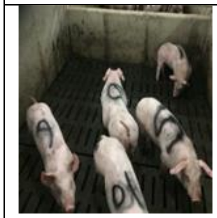	Magnified example of pigs with numbers on their backs to facilitate video-monitoring

Figure S2. Pig tail-biting scores.

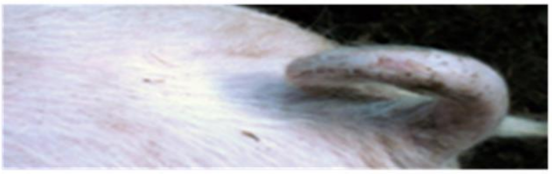	**Score 0:** No tail damage (no lesion)
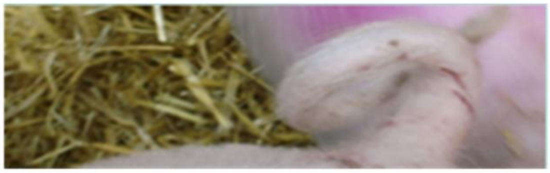	**Score 1:** Some scratches visible on the tail
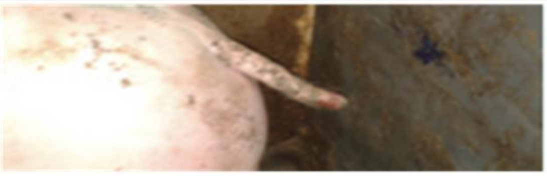	**Score 2:** A small bleeding lesion on the tail
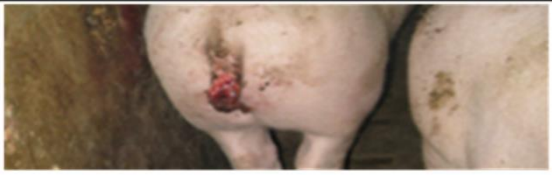	**Score 3:** A major bleeding lesion, including loss of tail

Adapted from Institut du porc, Institut national de la recherche agronomique. Prévenir la douleur chez le porc. (2016). Available online at: https://www.ifip.asso.fr/sites/default/files/pdf-documentations/dossier-prevention-douleur-porccaudectomie-caudophagie.pdf.

Figure S3. Non-metric multidimensional scaling (NMDS) Yue and Clayton index plot illustrating the comparison of intestinal microbiota of the ATB treated positive control group with the negative control pigs at t0 and t1.

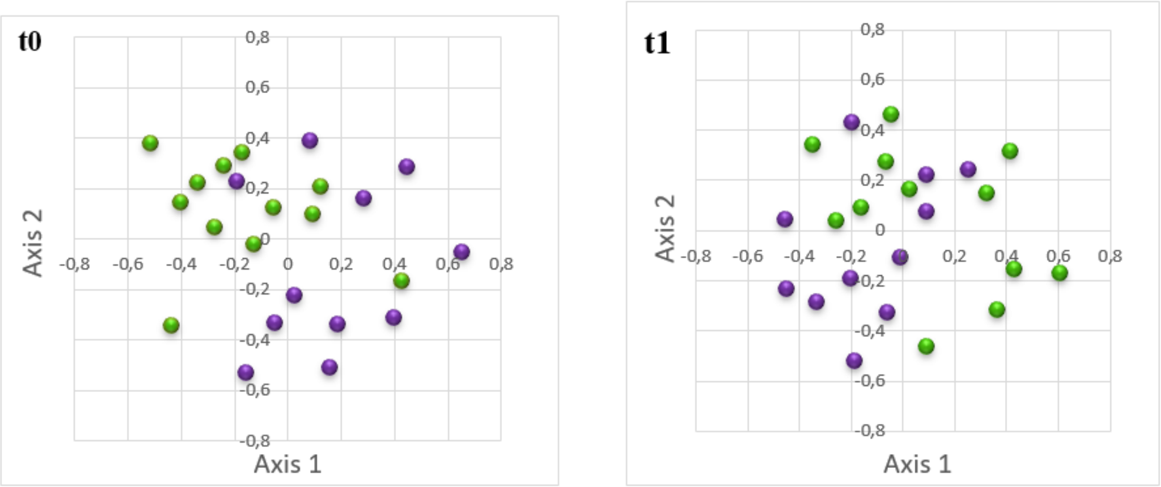

t0: time of the pig group formation; t1: four weeks after the pig group formation. Green: negative control pigs; purple: ATB-treated positive control group.

Table S1. Genera and OTUs associated with the biter pig group compared to the negative control pig group at t0.

GENERA	Taxonomy	Group	LDA	*p*Value
Coprococcus	*B.F.C.Clostridiales.Lachnospiraceae*	biter	3.30	0.0163
Clostridium_IV	*B.F.C.Clostridiales.Ruminococcaceae*	biter	2.69	0.0312
Lactobacillus	*B.F.Bli.Lactles.Lactctobacillaeae*	N. control	4.47	0.0031
Butyricicoccus	*B.F.C.Cles.Bacteroidales*	N. control	3.54	0.0363
Pseudobutyrivibrio	*B.F.C.Cles..Lachnospiraceae*	N. control	2.54	0.0163
**OTUs**	**Taxonomy**	**Group**	**LDA**	***p*Value**
Otu00020	*B.Btes.Bacteroidetes_ucld*	biter	4.03	0.0097
Otu00069	*B.Firmicute_ucld*	biter	3.41	0.0007
Otu00045	*B.F.C.Cles.Lachnoe.Coprococcus*	biter	3.25	0.0163
Otu00096	*B.Btes.Bdia.Bles.Prceae.Prevotella*	biter	3.01	0.0476
Otu01288	*B.F.C.Cles.Ruminoc.Faecalibacterium*	biter	2.86	0.0404
Otu00128	*B.F.C.Cles.Lachno.Blautia*	biter	2.79	0.0363
Otu00027	*B.Btes.Btes_ucld*	biter	2.77	0.0116
Otu00139	*B.Btes.Bdia.Bles.Prceae.Alloprevotella*	biter	2.72	0.0363
Otu00107	*B.Btes.Btes_ucld*	biter	2.70	0.0263
Otu01045	*Clostridiales_ucld*	biter	2.66	0.0036
Otu01146	*Clostridia_ucld*	biter	2.65	0.0404
Otu01361	*B.Btes.Bdia.Bles.Prceae.Prevotella*	biter	2.63	0.0414
Otu00975	*Ruminococcaceae_ucld*	biter	2.61	0.0113
Otu00797	*Clostridiales_ucld.*	biter	2.60	0.0404
Otu01377	*B.Firmicutes_ucld*	biter	2.60	0.0404
Otu01071	*Clostridiales_ucld*	biter	2.58	0.0299
Otu01557	*Lachno_ucld*	biter	2.54	0.0404
Otu00169	*Clostridiales_ucld*	biter	2.54	0.0011
Otu00152	*B.Btes.Bdia.Bles.Prceae.Prevotella*	biter	2.54	0.0225
Otu00797	*Clostidiales_ucld*	biter	2.52	0.0404
Otu01506	*Bacteroidale_ucld*	biter	2.50	0.0404
Otu01628	*B.Btes.Bdia.Bles.Prceae.Prevotella*	biter	2.49	0.0404
Otu00002	*B.F.Bacilli.Lactles.Lactceae.Lactobacillus*	N. control	4.51	0.0016

Otu00016	*B.F.C.Cles.Lachno.Cellulosilyticum*	N. control	3.94	0.0056
Otu00037	*B.Btes.Bdia.Bles.Prceae.Prevotella*	N. control	3.42	0.0163
Otu00142	*Actinobacterium_ucld*	N. control	3.17	0.0193
Otu00164	*Ruminococcaceae_ucld*	N. control	3.02	0.0163
Otu00132	*B.F.C.Cles.Peptococcaceae_1.Peptococcus*	N. control	2.86	0.0488
Otu01257	*B.F.C.Cles.Ruminoc.Ruminococcaceae_ucld*	N. control	2.77	0.0254
Otu00245	*B.Btes.Bdia.Bles.Prceae.Prevotella*	N. control	2.76	0.0422
Otu01360	*B.F.C.Clostridiales_ucld*	N. control	2.76	0.0105
Otu01975	*B.F.C.Cles.Lachno.Dorea*	N. control	2.76	0.0254
Otu01968	*Porphyromonadaceae_ucld*	N. control	2.73	0.0254
Otu00267	*B.Btes.Bdia.Bles.Prceae.Paraprevotella*	N. control	2.71	0.0362
Otu02401	*Ruminococcaceae_ucld*	N. control	2.70	0.0254
Otu01725	*B.Btes.Bdia.Bles.Prceae.Prevotella*	N. control	2.62	0.0141
Otu00258	*B.Oxalobacteraceae_ucld*	N. control	2.61	0.0080
Otu00241	*B.Prote.Gprot.Aerles.Suceae.Succinivibrio*	N. control	2.61	0.0312
Otu00181	*B.Spiro.Spirotia.Spiroles.Spiroceae.Treponema*	N. control	2.58	0.0116
Otu00175	*B.F.C.Cles.Lachno.Pseudobutyrivibrio*	N. control	2.54	0.0163
Otu00159	*B.F.C.Cles.Ruminoc.Oscillibacter*	N. control	2.54	0.0488

Table S2. Genera and OTUs associated with the biter pig group compared to the negative control pig group at t1.

GENERA	Taxonomy	Group	LDA	*p*Value
Roseburia	*B.F.C.Cles.Lachnospiraceae*	N. control	3.23	0.0422
Anaeroplasma	*Anaeroplasmataceae.Anaeroplasma*	N. control	2.91	0.0422
**OTUs**	**Taxonomy**	**Group**	**LDA**	***p*Value**
Otu00051	*B.F.C.Cles.Lachnospiraceae_ucld*	N. control	3.46	0.0193
Otu00075	*B.Btes.Bdia.Bacteroidales_ucld*	N. control	3.39	0.0004
Otu00035	*B.F.C.Clostridiales_ucld*	N. control	3.23	0.0422
Otu00114	*B.F.C.Cles.Lachnospiraceae_ucld*	N. control	2.89	0.0312
Otu00093	*B.Bacteroidetes_ucld*	N. control	2.79	0.0116
Otu00149	*B.F.C.Clostridiales_ucld*	N. control	2.69	0.0266
Otu00160	*B.F.C.Cles.Ruminococcaceae_ucld*	N. control	2.64	0.0193
Otu00267	*B.Btes.Bdia.Bles.Prceae.Paraprevotella*	N. control	2.64	0.0422
Otu00210	*B.Btes.Bdia.Bles.Porphyromonadaceae_ucld*	N. control	2.50	0.0267

Table S3. Genera and OTUs associated with the bitten pig group compared to the negative control pig group at t0.

GENERA	Taxonomy	Group	LDA	*p*Value
Sphaerochaeta	*B.Spt.Sptia.Sptales.Sptceae*	bitten	3.21	0.0056
Blautia	*B.F.C.Cles.Lachnospiraceae*	bitten	2.96	0.0312
Lactobacillus	*B.F.Bacilli.Lactobacillales.Lactobacillaceae*	N. control	4.57	0.0068
Intestinimonas	*B.F.C.Cles.Ruminoc*	N. control	3.38	0.0363
**OTUs**	**Taxonomy**	**Group**	**LDA**	***p*Value**
Otu00010	*B.F.Nega.Sel8moles.Ac.Phascolarctobacterium*	bitten	3.61	0.0487
Otu00027	*B.Btes.Btes_ucld.Btes_ucld.Btes_ucld.Btes_ucld*	bitten	3.26	0.0138
Otu00104	*B.Btes.Bdia.Bles.Bles_ucld.Bles_ucld*	bitten	3.18	0.0068
Otu01288	*B.F.C.Cles.Ruminoc.Faecalibacterium*	bitten	3.17	0.0405
Otu00174	*B.Spt.Sptia.Sptales.Sptceae.Sphaerochaeta*	bitten	3.16	0.0423
Otu01451	*B.Btes.Bdia.Bles.Porphy.Parabacteroides*	bitten	2.90	0.0191
Otu00069	*B.F.F_ucld.F_ucld.F_ucld.F_ucld*	bitten	2.87	0.0068
Otu01334	*B.ActinoB.ActinoB.CorioBles.CorioBceae.ucld*	bitten	2.85	0.0405
Otu00128	*B.F.C.Cles.Lachnospiraceae.Blautia*	bitten	2.71	0.0267
Otu00950	*B.Btes.Bdia.Bles.prevceaee.Alloprevotella*	bitten	2.70	0.0405
Otu00152	*B.Btes.Bdia.Bles.prevceaee.Prevotella*	bitten	2.69	0.0132
Otu00169	*B.F.C.Cles.Cles_ucld.Cles_ucld*	bitten	2.69	0.0228
Otu00950	*B.Btes.Bdia.Bles.prevceaee.Alloprevotella*	bitten	2.67	0.0405
Otu01231	*B.F.C.Cles.Ruminoc.Ruminoc_ucld*	bitten	2.62	0.0405
Otu01557	*B.F.C.Cles.Lachnospiraceae.ucld*	bitten	2.55	0.0405
Otu00182	*B.F.C.Cles.Cles_ucld.Cles_ucld*	bitten	2.52	0.0228
Otu00266	*B.F.C.Cles.Lachnospiraceae.Blautia*	bitten	2.52	0.0228
Otu00002	*B.F.Bacilli.Lactles.Lactceae.Lactobacillus*	N. control	4.58	0.0031
Otu00016	*B.Btes.Bdia.Bles.Bles_ucld.Bles_ucld*	N. control	4.03	0.0011
Otu00054	*B.Btes.Bdia.Bles.prevceaee.Prevotella*	N. control	3.59	0.0423
Otu00090	*B.F.C.Cles.Ruminoc.Intestinimonas*	N. control	3.38	0.0192
Otu01257	*B.F.C.Cles.Ruminoc.Ruminoc_ucld*	N. control	2.62	0.0254
Otu00181	*B.Spt.Sptia.Sptales.Sptceae.Treponema*	N. control	2.59	0.0116
Otu01725	*B.Btes.Bdia.Bles.prevceaee.Prevotella*	N. control	2.59	0.0359
Otu00258	*B.ProteoB.BetaproteoB.Burkles.Oxalo. ucld*	N. control	2.55	0.0228
Otu01975	*B.F.C.Cles.Lachnospiraceae.Dorea*	N. control	2.51	0.0254

Table S4. Genus and OTUs associated with the bitten pig group compared to the negative control pig group at t1.

GENUS	Taxonomy	Group	LDA	*p*Value
Alistipes	*B.Btes.Bdia.Bles.Rikenellaceae*	bitten	2.91	0.036
**OTUs**	**Taxonomy**	**Group**	**LDA**	***p*Value**
Otu00021	*B.Btes.Bdia.Bles.Prevotellaceae.Prevotella*	bitten	3.52	0.019
Otu00174	*B.Sptes.Sptia.Sples.Spceae.Sphaerochaeta*	bitten	3.01	0.042
Otu00029	*B.Btes.Bdia.Bles.Prevotellaceae.Prevotella*	bitten	2.95	0.042
Otu00157	*B.F.C.Cles.Cles_ucld.Cles_ucld*	bitten	2.78	0.019
Otu00200	*B.Bacteroidetes_ucld*	bitten	2.53	0.036
Otu00001	*B.Btes.Bdia.Bles.Prevotellaceae.Prevotella*	N. control	3.90	0.022
Otu00054	*B.Btes.Bdia.Bles.Prevotellaceae.Prevotella*	N. control	3.37	0.011
Otu00121	*B.Btes.Bdia.Bles.Prevotellaceae.Prevotellaceae_ucld*	N. control	2.82	0.036
Otu00231	*B.Btes.Bdia.Bles.Porphyceae.Porphy_ucld*	N. control	2.55	0.013
Otu00189	*B.F.C.Cles.Ruminoc.Ruminococcaceae_ucld*	N. control	2.51	0.036

Abbreviations for Tables S1-S4:

B: Bacteria; Btes: Bacteroidetes; Bdia: Bacteroidia; Bles: Bacteroidales; F: Firmicute; C: Clostridia; Cles: Clostridiales; Ruminoc: Ruminococcaceae ; Nega: Negativicutes; Spt: Spirochaetes; Sptia: Spirochaetia; Sptales: Spirochaetales; Sptceae: Spirochaetaceae; Lachno: Lachnospiraceae; Proteob: Proteobacteria; Betapb: Betaproteobacteria; Burkles: Burkholderiales; Oxal: Oxalobacteraceae; prevceae: Prevotellaceae; Ac: acidaminococcaceae; Porphy: Porphyromonadaceae; ucld: unclassified; N. control: Negative control; LDA: Linear Discriminant Analysis; t0: time of pig group selection; t1: four weeks following selection.
